# The Correlation Between Body Mass Index and Computed Tomography Angiography on Vascular Positioning in Anterolateral Thigh Flap Transplantation

**DOI:** 10.5334/jbsr.2762

**Published:** 2022-11-08

**Authors:** Yan Jiang, Qilong Chen, Wentao Xu, Min Cao

**Affiliations:** 1 Department of Radiology, Wuxi 9th People’s Hospital Affiliated to Soochow University, Wuxi, China

**Keywords:** anterolateral thigh perforator flap, computed tomography angiography, body mass index, region of interest, blood vessel

## Abstract

**Objective:**

This work aimed to investigate the correlation between body mass index (BMI) and computed tomography angiography (CTA) in the vascular positioning of anterolateral thigh perforator flap (ALT) and to discuss the influence of CTA on the vascular positioning of the ALT by taking BMI as a variable.

**Methods:**

A total of 117 patients who admitted to our hospital (Wuxi Ninth People’s Hospital) from January 2017 to May 2019, hospitalized due to limb injury and needed ALT transplantation were collected in this work. According to the BMI, the patients were divided into group A (BMI < 18.5) with 18 cases, group B (18.5 ≤ BMI < 24) with 56 cases, and group C (BMI ≥ 24) with 43 cases. CTA was used to locate the perforator vessels in anterolateral thigh (ALT) flap of the three groups. All surgical and CTA data were collected.

**Results:**

There was a significant positive correlation between BMI and CTA positioning (P < 0.05).

**Conclusion:**

The larger the BMI, the more accurate the CTA positioning. When the BMI was not less than 18.5, CTA positioning should be the most accurate.

## Introduction

The anterolateral thigh (ALT) flap is a versatile soft tissue flap that can be harvested as a fasciocutaneous or myocutaneous flap. Vascularized fascia can be included or the pedicle may be harvested as a flow-through flap [1]. The flap can also be harvested incorporating multiple skin islands or incorporating separate skin and muscle components as a chimeric flap [2]. According to the clinical research, the ALT flap is known as the “universal flap” [3] and is the first choice for wound repair. When a large flap is needed, the entire lateral thigh can be harvested by combining the ALT with either the tensor fascia lata or the anteromedial thigh flap to obtain a conjoined flap [4].

The purported difficulty with the use of this flap lies in the anatomical variations that may render this flap unreliable. Later in 2003, Wei Fuquan et al. proposed a new definition of “free-style free flap” [5]. Once a perforator is found, a vascular pedicle of sufficient diameter and length can be traced to form a flap. In modern skin graft surgery, the perforator positioning technology provides powerful help for the design of the surgical area. By the accurate positioning of the perforator, a suitable donor- site shape can be designed according to the shape of the receiving area [6]. The imaging examination plays an important role in the preoperative positioning of the perforator. At present, the widely used and recognized perforator positioning method is computed tomography angiography (CTA) [7]. During CTA examination, different body mass index (BMI) may affect the precision of positioning. In this work, retrospective analysis was given to the patients undergoing ALT flap transplantation in our hospital. The correlation between BMI and CTA on vascular positioning of anterolateral thigh perforator flap (ALT) was explored.

## Patients and Methods

This work had been approved by the Research Ethics Committee.

The patients who underwent ALT flap transplantation from January 2017 to June 2019 in our hospital were retrospectively included in this work. A total of 117 inpatients were included and they all underwent CTA examination. Among them, 39 were females and 78 were males. The oldest patient was 72 years old and the youngest patient was 14 years old with an average age of 43.58 ± 12.53 years. Imaging data and surgical data corresponding to each patient were collected. The commonly used BMI calculation method was weight (kg) divided by the square of height (m^2^) that was, BMI = weight/(height*height) (kg/m^2^). According to the “Guidelines for the Prevention and Control of Adult Overweight and Obesity in China” in April 2003, the 117 patients were divided into three groups: Underweight group (group A), with BMI < 18.5 and including 18 cases; normal group (group B), with 18.5 ≤ BMI < 24 and including 56 cases; overweight group (group C), with BMI ≥ 24 and including 43 cases. All the three groups underwent CTA examinations before surgery with CTA contrast agent of Onipak (350 mgI/mL).

The patient adopted a standard posture in the supine position. The line from the donor site’s anterior superior iliac spine to the lateral edge of the patella was taken as the Y-axis and the vertical line at the midpoint of the former line was the X-axis. This midpoint position was used as the reference point for the positioning of the perforator flap. A small cotton ball dipped in diluted iohexol was placed at this point to fix it. Smart detection was used to trigger the scan mode. The monitoring layer was selected at the level of the lower part of the abdominal aorta (1 cm above the bifurcation of the common iliac artery (IA) on both sides). The CT value of the abdominal aorta at this level was taken as the reference value (ROI, area 10 mm^2^). When the CT value in the ROI reached the threshold (150 HU), the scan was triggered manually. Scanning range was 10 mm from the upper anterior superior iliac spine of both lower limbs to 10 mm from the lower edge of the patella. The reconstructed original data of 1 mm layer thickness was pushed to the GE AW Server 4.7 post-processing workstation for further image processing.

**Figure 1:**
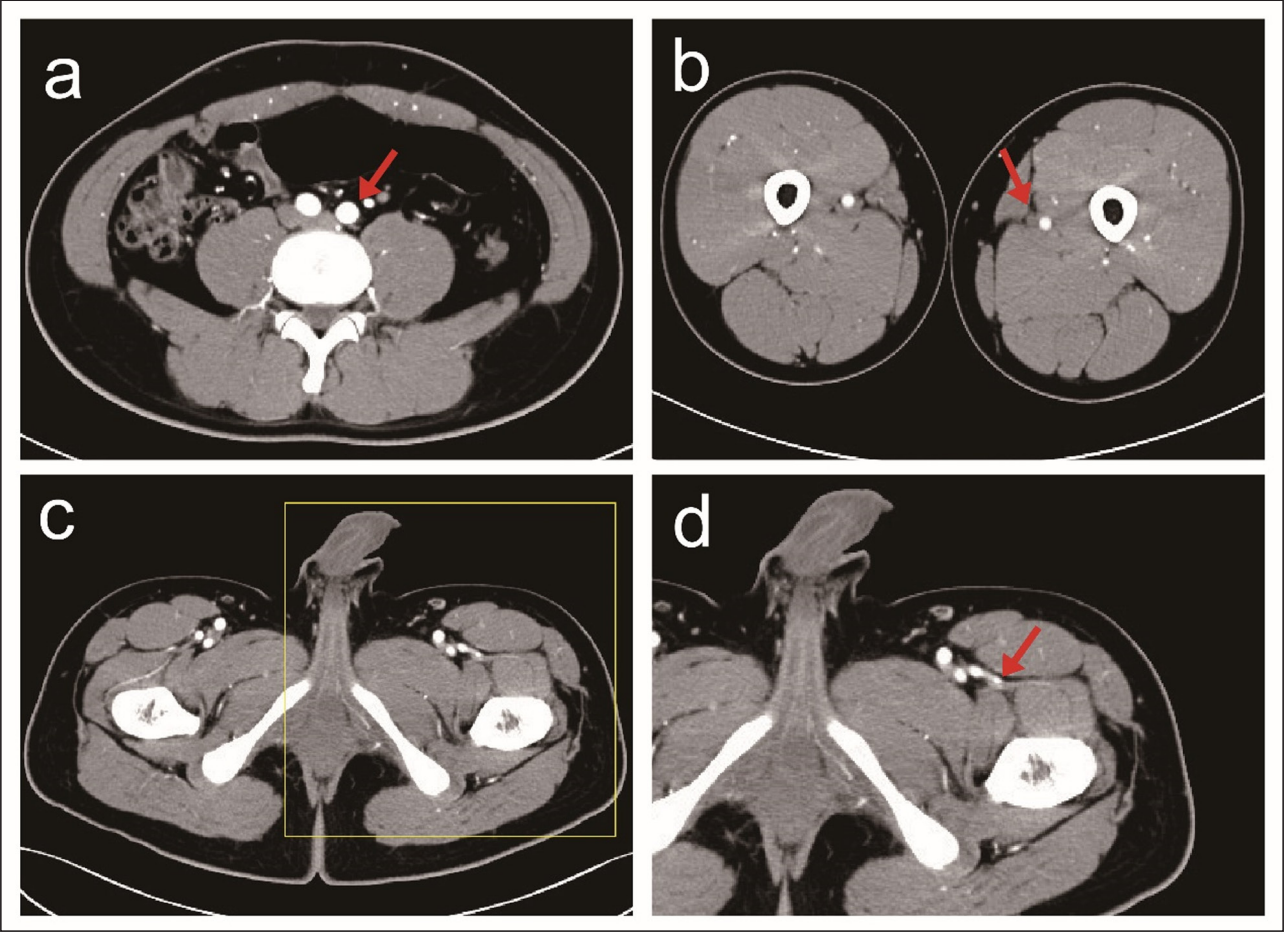
CTA Data Processing. **a.** The abdominal aorta bifurcation level; **b.** The mid-level of the left FA; **c.** The first perforating branch of the descending branch of the left lateral circumflex femoral artery (CFA), penetrated by 1 cm; **d.** The first perforating branch of the descending branch of the left lateral CFA, with 10 times magnified.

**Figure 2:**
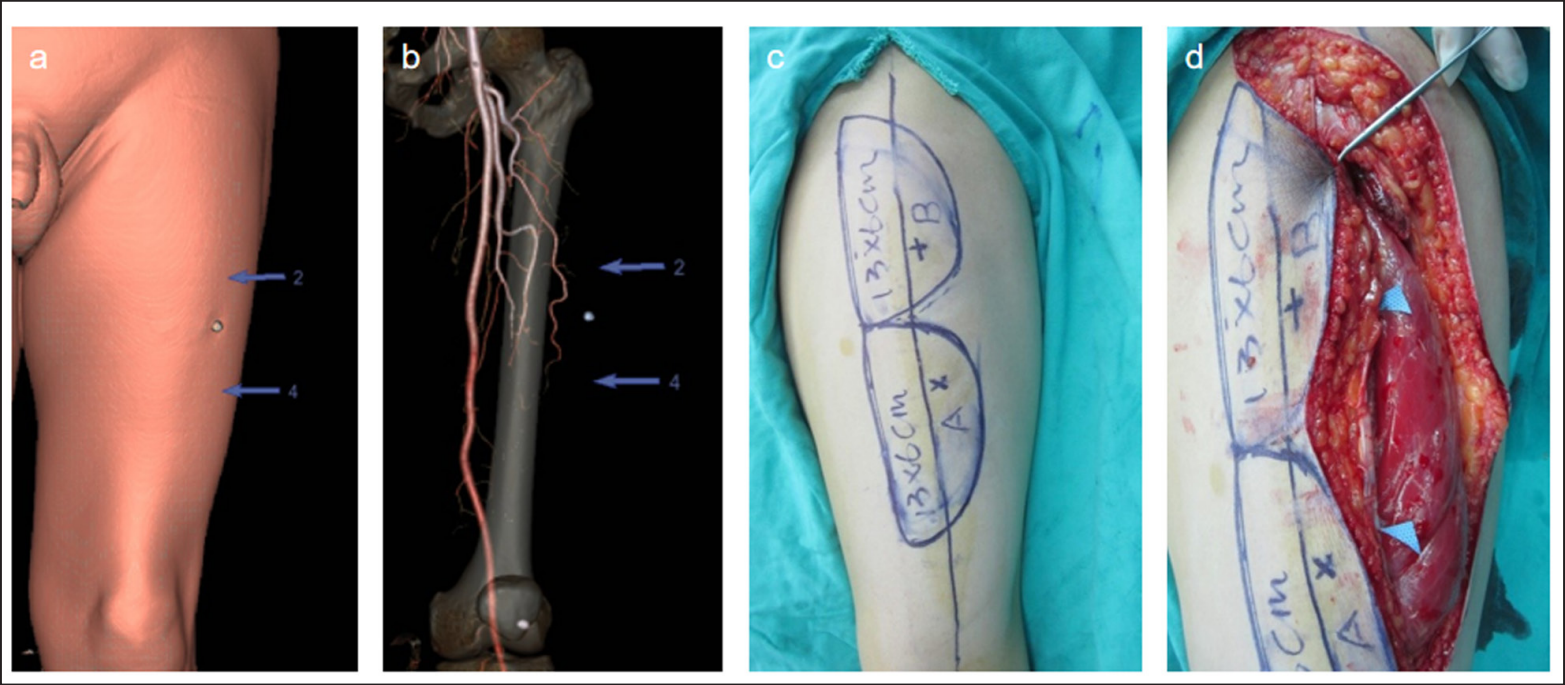
Comparison between preoperative and intraoperative body surface simulation positioning of CTA. **a.** Simulation and positioning of the body table; **b.** VR projection of perforating vessels; **c.** Preoperative surgical plan design; **d.** Intraoperative positioning of perforator.

To objectively evaluate the image quality of patients, several parameters were selected for analysis (Figure 1): (1) The three-level image (the level of the bifurcation of the abdominal aorta and the common (IA), the level of the mid-section of the left femoral artery (FA) and the level of the descending branch of the left lateral (CFA) was selected from the original cross-sectional image. Then analysis was conducted to determine whether the background noise of different BMI images had a substantial influence on the diagnostic image. (2) The level of the bifurcation of the abdominal aorta common IA, the level of the left FA, and the level of the left lateral CFA of the descending branch (in which the first perforating branch penetrated about 1 cm as the detection level) were taken and the ROIs at the center of the artery were selected, with areas of 0.1 cm^2^, 0.1 cm^2^, and 0.01 cm^2^. Due to the short diameter of the perforator, the CT value was measured after being magnified by 10 times appropriately and the measurement area was about 0.5 mm away from the blood vessel wall. (3) The soft tissues of the same three (the soft tissue of bifurcation of the abdominal aorta common IA, SIA; the soft tissue of the mid-section of the left FA, SFA; and the soft tissue the left lateral CFA, SCFA) detection levels 1 cm away from the measured artery radius were taken as the reference points to measure the CT values. During the operation, a dominant perforator position in the grafted skin flap was selected as the standard point, and the errors of the CTA positioning points and the standard points in different BMI populations were compared (Figure 2).

SPSS21.0 software was used for statistical analysis of data and α = 0.05 was used as the test level for comparison between groups. The measurement data were compared between groups with two-sample t-test. Chi-square test was conducted to compare the counting data between groups. Variance F test was adopted for the comparison of measurement data between groups and LSD-T method was used for pairwise comparison between groups. The significance was set to the level of P < 0.05.

## Results

With the relevant data measured, an objective analysis was given to the images. Arterial SNR (signal-noise ratio) referred to the measurement of arterial CT value/background noise, soft tissue SNR referred to the measurement of soft tissue CT value/background noise, CNR (contrast-to-noise ratio, CNR) was calculated as (arterial CT value of measurement layer – CT value of soft tissue of measurement layer)/background noise. Table 1 exhibited the comparative analysis of the image characteristics of the three groups. There were no statistically notable differences in the three groups of related image quality control indicators.

**Table 1:** Image quality control related to the three.


VARIABLE		GROUP			P

		A	B	C	

BMI		17.61 ± 0.91	21.62 ± 1.22	22.76 ± 3.44	0.20

Gender(n)	Male	3	16	5	0.10

	Female	15	40	38	

Age		48.06 ± 15.94	42.80 ± 13.45	42.72 ± 9.16	0.25

CTA positioning		6.67 ± 1.94	5.50 ± 2.17	3.35 ± 1.31	0.001

IA		432.42 ± 88.17	426.67 ± 87.84	419.00 ± 81.99	0.82

SIA		65.99 ± 7.52	66.99 ± 6.83	63.95 ± 7.20	0.11

SNR_IA_		153.53 ± 22.58	155.61 ± 24.33	159.89 ± 29.36	0.61

SNR_SIA_		23.81 ± 3.80	24.77 ± 3.60	24.57 ± 4.28	0.66

CNR_IA_		129.73 ± 22.36	130.84 ± 23.26	135.32 ± 27.13	0.60

FA		152.83 ± 15.79	162.36 ± 13.73	160.02 ± 13.97	0.05

SFA		65.76 ± 6.30	67.76 ± 5.20	68.32 ± 6.36	0.29

SNR_FA_		52.04 ± 6.95	51.40 ± 7.68	51.45 ± 5.22	0.94

SNRSFA		22.39 ± 2.77	21.40 ± 2.59	22.00 ± 2.75	0.31

CNRFA		29.64 ± 6.05	30.00 ± 6.00	29.44 ± 4.82	0.89

CFA		463.78 ± 83.81	420.70 ± 68.01	434.35 ± 76.14	0.10

SCFA		65.00 ± 5.70	65.55 ± 3.67	67.31 ± 5.40	0.10

SNR_CFA_		156.46 ± 39.23	146.85 ± 31.66	155.46 ± 23.72	0.29

SNR_CFA_		22.07 ± 4.22	23.17 ± 5.56	24.39 ± 3.71	0.19

CNR_CFA_		134.39 ± 37.41	123.67 ± 28.09	131.07 ± 22.58	0.25


BMI, CTA positioning difference between CTA positioning and intraoperative positioning. IA, FA, and CFA referred to the average CT values of the three groups of blood vessels. SIA, SFA, and SCF referred to the average CT values of the three groups of para-arterial soft tissues. SNRIA, SNRFA, and SCFA, referred to SNR of the three groups of blood vessels. SNRSIA, SNRSFA, and SNRCFA referred to the soft tissue SNR of the three groups. CNRIA, CNRFA, and CNRCFA referred to CNRs of the three groups of arteries.

Total 117 subjects were investigated, including 93 males (accounting for 79.49%) and 24 females (accounting for 20.51%) and there was no statistically obvious difference in gender among the three groups(P = 0.10). The average age was 43.58 ± 12.53 years old, with the oldest age of 72 years old and the youngest age of 14 years old. There was no statistically notable difference in age among the three groups (P = 0.25). The average BMI was 22.76 ± 3.44 kg/m^2^, with the maximum BMI of 31.25 kg/m^2^ and the minimum BMI of 15.57 kg/m^2^, and there was no statistically remarkable difference in BMI among the three groups (P = 0.20). The Chi-square test indicated a statistically obvious difference in CTA positioning among the three groups (P = 0.001).

According to Pearson correlation analysis, there was an obvious correlation between BMI and CTA positioning (P < 0.05), as shown in Table 2. There was no statistically obvious difference between BMI and related image quality control indicators.

**Table 2:** Correlation analysis of BMI and CT values of arteries in various parts. There was no statistically obvious difference between BMI and related image quality control indicators. There was a correlation between BMI and CTA positioning.


VARIABLE	x̄ ± s	r	P

CAT positioning	4.89 ± 2.22	–0.61	0.001

IA	424.74 ± 81.99	0.01	0.95

SIA	65.71 ± 7.15	–0.13	0.18

SNRIA	156.86 ± 25.95	0.08	0.39

SNRSIA	24.55 ± 3.87	–0.04	0.67

CNRIA	132.32 ± 24.53	0.09	0.33

FA	160.03 ± 14.39	0.08	0.39

SFA	67.66 ± 5.84	0.08	0.37

SNRFA	51.51 ± 6.71	0.08	0.39

SNRSFA	21.78 ± 2.68	–0.04	0.68

CNRFA	29.74 ± 5.56	–0.03	0.74

CFA	432.34 ± 74.45	–0.05	0.58

SCFA	66.11 ± 4.75	0.13	0.14

SNRCFA	151.49 ± 30.41	0.04	0.66

SNRCFA	23.45 ± 4.78	0.14	0.12

CNRCFA	128.04 ± 27.98	0.02	0.83


**Figure 3:**
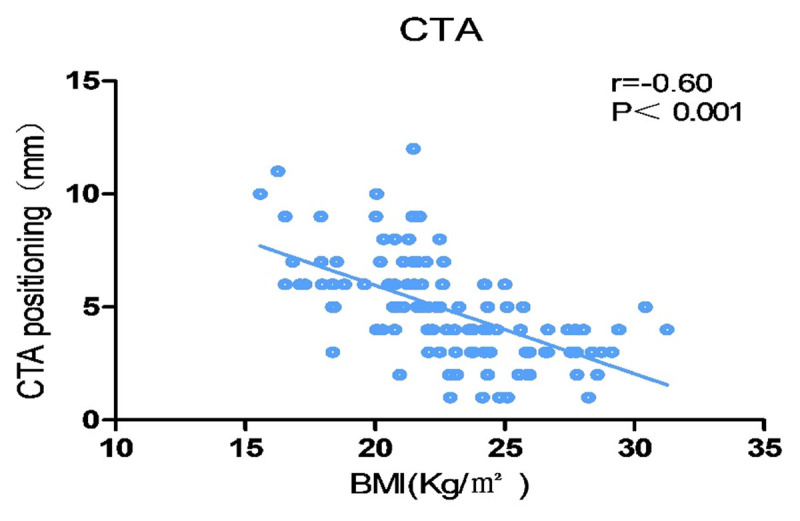
Correlation between BMI and CTA positioning. There was an obvious negative correlation between BMI and CTA positioning. The larger the BMI, the smaller the CTA positioning difference.

**Figure 4:**
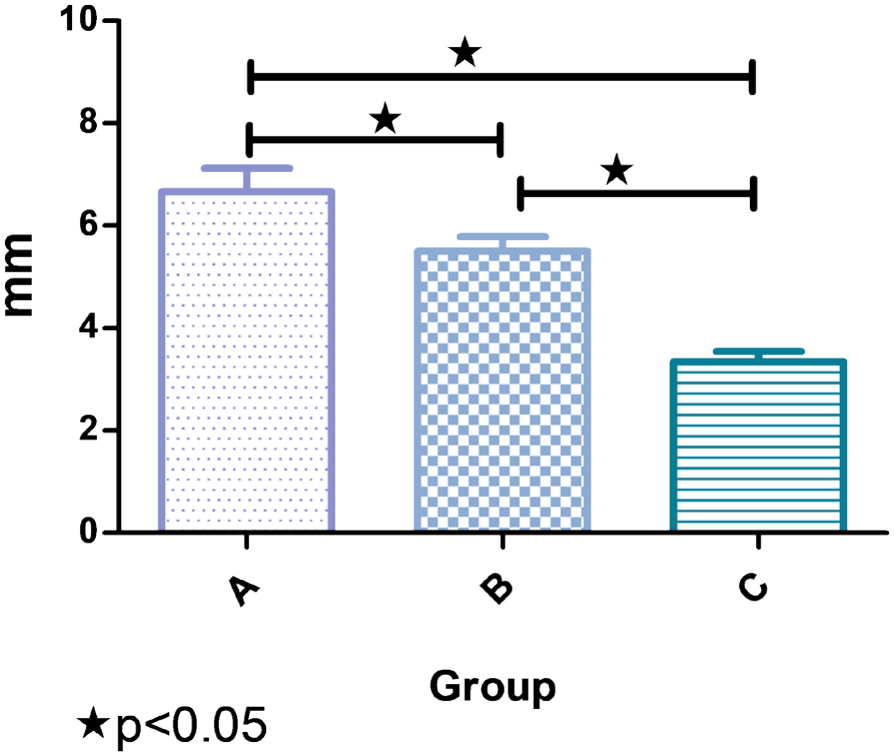
Comparison of CTA positioning of BMI in the three groups. There were notable differences in CTA positioning among the three groups.

There was an obvious negative correlation between BMI and CTA positioning (r < 0), as shown in Figure 3. The results of pairwise comparison among the three groups showed that the location difference of CTA in group A was greatly higher than that in group B and group C, namely, the larger the BMI the smaller the CTA positioning difference as shown in Figure 4 and Table 3.

**Table 3:** Comparison of CTA positioning of BMI in the three groups.


GROUP	CTA POSITIONING (mm)	t	P

A	6.667 ± 1.940	4.274	0.001

B	4.500 ± 2.166^a^	2.985	0.004

C	3.349 ± 1.307^ab^	8.892	0.000

F	20.344		

P	0.000		


Compared with group A, P < 0.05. Compared with group B, P < 0.05.

## Discussion

Since the late 1990s, major developments have been made in the design of skin flaps and the types of skin flaps have become more abundant. This is mainly due to the great development of perforator angiography technology [8,9]. With the continuous development of imaging technology, perforator flap transplantation technology has also been improved incessantly [10]. The imaging technology can better determine the flap transplantation plan, improve the precision of the operation, and shorten the time for the flap removal during the operation.

Nowadays, CTA technology has been widely used in various fields of perforator flap positioning surgery. At the present stage [11,12], multi-slice spiral CT with more than 64 slices is commonly used and the thinnest slice thickness is as thin as 0.625 mm. It can present a three-dimensional image and the whole image is more intuitive [13,14]. Meanwhile, it can accurately exhibit the diameters of blood vessels and rami perforantes as well as the small perforators. Thus, the surgeons can perform perforator flap surgery on this basis with the precision and safety of rami perforantes greatly improved [15].

Several researches have indicated the high stability of positioning by CTA before the percutaneous valve graft surgery and there is no major error due to the differences in medical institutions or operators [16,17]. This work presented that there was an obvious negative correlation between BMI and CTA positioning, namely, the larger the BMI value the more accurate the CTA positioning. By comparing with the three groups of axial films located by CTA, it was found that the thickness of the local subcutaneous fat layer was uneven and the perforator branch penetrated the deep fascia. In group C, the positions where the perforator branch penetrated the superficial fascia layer and the end penetrated the skin surface were determined, providing strong support for the special ultra-thin skin flap.

However, during the CTA examination, a contrast agent needs to be injected into the patient’s body which may have adverse effects on the patient’s kidneys. The contrast agent may hurt the patient’s blood vessels and cause vasospasm [18], which can affect the accuracy of the assessment of small-caliber blood vessels [19]. Allergies may also be caused and it needs to pay attention to the radiation dose issues [20]. There were also some shortcomings in this work. For example, it was difficult to measure the inner diameter of blood vessels in CTA examination. Coordinate axes were needed to set reference points to locate the body surface and the measurement data might change along with the patient’s position change during the process from the simulated positioning of skin surface to the marking of donor site. With the development of precision treatment in the field of microsurgery, the precise positioning of the human body by CTA shall become an indispensable part in the field of microsurgery [21].
